# Supplementary material for: Cohort profile: The ENTWINE iCohort study, a multinational longitudinal web-based study of informal care
Source: PLoS One. 2024 Jan 18;19(1):e0294106. doi: 10.1371/journal.pone.0294106 (PMC10796045; doi:10.1371/journal.pone.0294106)
Supplement: S1 Table — (DOCX) [file pone.0294106.s001.docx]

| **S1 Table. A complete list of all caregiver organisations and advocacy groups involved in the recruitment of participants.** | |
| --- | --- |
| **Organisation/Group** | **Country** |
| Allianz pflegende Angehörige | Germany |
| Anziani e non solo | Italy |
| Associazione C’ENTRO | Italy |
| Associazione de Banfield | Italy |
| Federazione Alzheimer Italia | Italy |
| Caffè Alzheimer Falconara | Italy |
| Associazione PAzienti LIberi dalle Neoplasie UROteliali (PALINURO) | Italy |
| Diabete Italia Onlus | Italy |
| Associazione Parkinson Marche | Italy |
| Rete Caregiver | Italy |
| Associazione Errante | Italy |
| ASPI Groane | Italy |
| Caregivers Israel | Israel |
| Camoni - Friends for Health | Israel |
| Think Bodywhys | Ireland |
| Family caregivers Ireland | Ireland |
| Shine | Ireland |
| Saint Vincent de Paul Ireland | Ireland |
| Brain Tumour Ireland | Ireland |
| Care Alliance Ireland | Ireland |
| Chronic Pain Ireland | Ireland |
| Muscular Dystrophy Ireland | Ireland |
| Polio Survivors Ireland | Ireland |
| Inclusion Ireland | Ireland |
| Fundacja Hospicyjna | Poland |
| Stowarzyszenie Pomocy Psychologicznej Syntonia | Poland |
| Fundacja SM – walcz o siebie | Poland |
| Polskie Stowarzyszenie Diabetyków | Poland |
| Polskie Stowarzyszenie Pomocy Osobom z Chorobą Alzheimera | Poland |
| Fundacja W Związku Z Rakiem | Poland |
| Stowarzyszenie UNICORN | Poland |
| Fundacja ORCHidea | Poland |
| Niepelnosprawni.pl | Poland |
| Fundacja Instytut Praw Pacjenta i Edukacji Zdrowotnej | Poland |
| Punkt Wsparcia Seniora | Poland |
| Centrum Wsparcia – opiekunów nieformalnych i faktycznych | Poland |
| MantelzorgNL | the Netherlands |
| Zorgbelang | the Netherlands |
| Vilans | the Netherlands |
| Movisie | the Netherlands |
| Anhörigas riksförbund | Sweden |
| Hjärt-Lungfonden | Sweden |
| Diabetesfonden | Sweden |
| Prostatacancerförbundet | Sweden |
| Sarkomföreningen | Sweden |
| Alzheimerfonden | Sweden |
| Demenscentrum | Sweden |
| ParkinsonFonden | Sweden |
| AbbVie Sverigem | Sweden |
| ParkinsonFörbundet | Sweden |
| Anhörigstöd i Stockholms Län | Sweden |
| Parkinson Skåne | Sweden |
| Föreningen Balans Stockholm | Sweden |
| Svenska Ödemförbundet | Sweden |
| Mustaschkampen | Sweden |
| Alzheimerfonden | Sweden |
| Demensförbundet | Sweden |
| Anhörigas Riksförbund | Sweden |
| Svenska Diabetesförbundet | Sweden |
| Riksförbundet Balans | Sweden |
| Carers Wales | the United Kingdom |
| Carers Trust | the United Kingdom |
| Carers Officers Learning Improvement Network for Wales | the United Kingdom |
| Shared Lives | the United Kingdom |
| Carers Outreach Service | the United Kingdom |
| North East Wales Carers Information Service (NEWCIS) | the United Kingdom |
| Mencap Cymru | the United Kingdom |
| Age Cymru | the United Kingdom |
| Gwynedd Council | the United Kingdom |
| Centre for Ageing and Dementia Research | the United Kingdom |
| North Wales Social Care and Wellbeing Services Improvement Collaborative | the United Kingdom |
| Shared Care Scotland | the United Kingdom |
| Centre for International Research on Care | the United Kingdom |
| Labour and Equalities (CIRCLE) | the United Kingdom |
| Carers Federation | the United Kingdom |
| Join Dementia Research | the United Kingdom |
| Headway - The Brain Injury Association | the United Kingdom |
| The Brain Charity | the United Kingdom |
| MS Society UK | the United Kingdom |
| Breast Cancer UK | the United Kingdom |
