# Supplementary material for: Cohort profile: The ENTWINE iCohort study, a multinational longitudinal web-based study of informal care
Source: PLoS One. 2024 Jan 18;19(1):e0294106. doi: 10.1371/journal.pone.0294106 (PMC10796045; doi:10.1371/journal.pone.0294106)
Supplement: S2 Table — (DOCX) [file pone.0294106.s002.docx]

| **S2 Table. Care recipient condition(s) at baseline as reported by their caregiver.** | | |
| --- | --- | --- |
| **Condition** |  | **N = 1,731** |
| **Cardiological condition (e.g., heart attack, myocardial infarction, coronary thrombosis, congestive heart failure, etc.) = Yes, n (%)** |  | 293 (16.9%) |
| **Hypertension = Yes, n (%)** |  | 455 (26.3%) |
| **High blood cholesterol = Yes, n (%)** |  | 238 (13.7%) |
| **A stroke or cerebral vascular disease = Yes, n (%)** |  | 277 (16.0%) |
| **Diabetes = Yes, n (%)** |  | 291 (16.8%) |
| **Chronic lung disease (e.g., chronic bronchitis, emphysema, etc.) = Yes, n (%)** |  | 165 (9.5%) |
| **Cancer = Yes, n (%)** |  | 271 (15.7%) |
| **Gastrointestinal ulcer = Yes, n (%)** |  | 32 (1.8%) |
| **Parkinson disease = Yes, n (%)** |  | 159 (9.2%) |
| **Cataract(s) = Yes, n (%)** |  | 180 (10.4%) |
| **Hip fracture = Yes, n (%)** |  | 91 (5.3%) |
| **Other fractures = Yes, n (%)** |  | 78 (4.5%) |
| **Cognitive or memory disorders (e.g., Alzheimer's disease, dementia, etc.) = Yes, n (%)** |  | 518 (29.9%) |
| **Multiple sclerosis = Yes, n (%)** |  | 35 (2.0%) |
| **Rheumatoid Arthritis = Yes, n (%)** |  | 95 (5.5%) |
| **Osteoarthritis, or other rheumatism = Yes, n (%)** |  | 178 (10.3%) |
| **Chronic kidney disease = Yes, n (%)** |  | 97 (5.6%) |
| **Traumatic brain injury = Yes, n (%)** |  | 43 (2.5%) |
| **HIV/AIDS = Yes, n (%)** |  | 2 (0.1%) |
| **Other unspecified chronic condition(s) = Yes, n (%)** |  | 563 (32.5%) |
| **Number of diagnosed chronic conditions(s), n (%)** |  |  |
| *Care recipients with no diagnosed chronic condition* |  | 62 (3.6%) |
| *Care recipients with one diagnosed chronic condition* |  | 703 (40.6%) |
| *Care recipients with two or more diagnosed chronic conditions* |  | 966 (55.8%) |
| The number of missing values for all items is 141. | | |
