# Supplementary material for: Cohort profile: The ENTWINE iCohort study, a multinational longitudinal web-based study of informal care
Source: PLoS One. 2024 Jan 18;19(1):e0294106. doi: 10.1371/journal.pone.0294106 (PMC10796045; doi:10.1371/journal.pone.0294106)
Supplement: S3 Table — (DOCX) [file pone.0294106.s003.docx]

| **S3 Table. Care recipient condition(s) at baseline.** | | |
| --- | --- | --- |
| **Condition** |  | **N = 387** |
| **Cardiological condition (e.g., heart attack, myocardial infarction, coronary thrombosis, congestive heart failure, etc.) = Yes, n (%)** |  | 41 (10.6%) |
| **Hypertension = Yes, n (%)** |  | 107 (27.6%) |
| **High blood cholesterol = Yes, n (%)** |  | 72 (18.6%) |
| **A stroke or cerebral vascular disease = Yes, n (%)** |  | 31 (8.0%) |
| **Diabetes = Yes, n (%)** |  | 68 (17.6%) |
| **Chronic lung disease (e.g., chronic bronchitis, emphysema, etc.) = Yes, n (%)** |  | 64 (16.5%) |
| **Cancer = Yes, n (%)** |  | 73 (18.9%) |
| **Gastrointestinal ulcer = Yes, n (%)** |  | 14 (3.6%) |
| **Parkinson disease = Yes, n (%)** |  | 23 (5.9%) |
| **Cataract(s) = Yes, n (%)** |  | 27 (7.0%) |
| **Hip fracture = Yes, n (%)** |  | 8 (2.1%) |
| **Other fractures = Yes, n (%)** |  | 20 (5.2%) |
| **Cognitive or memory disorders (e.g., Alzheimer's disease, dementia, etc.) = Yes, n (%)** |  | 8 (2.1%) |
| **Multiple sclerosis = Yes, n (%)** |  | 29 (7.5%) |
| **Rheumatoid Arthritis = Yes, n (%)** |  | 28 (7.2%) |
| **Osteoarthritis, or other rheumatism = Yes, n (%)** |  | 84 (21.7%) |
| **Chronic kidney disease = Yes, n (%)** |  | 15 (3.9%) |
| **Traumatic brain injury = Yes, n (%)** |  | 11 (2.8%) |
| **HIV/AIDS = Yes, n (%)** |  | 5 (1.3%) |
| **Other unspecified chronic condition(s) = Yes, n (%)** |  | 188 (48.6%) |
| **Number of diagnosed chronic conditions(s), n (%)** |  |  |
| *No diagnosed chronic condition* |  | 8 (2.1%) |
| *One diagnosed chronic condition* |  | 159 (41.1%) |
| *Two or more diagnosed chronic conditions* |  | 220 (56.8%) |
| The number of missing values for all items is 15. | | |
